# Supplementary material for: Association between Lifestyle Modification and All-Cause, Cardiovascular, and Premature Mortality in Individuals with Non-Alcoholic Fatty Liver Disease
Source: Nutrients. 2024 Jun 28;16(13):2063. doi: 10.3390/nu16132063 (PMC11243540; doi:10.3390/nu16132063)
Supplement: Supplementary file 1 [file nutrients-16-02063-s001.zip › Supplementary materials/Table S1.docx]

**Table S1** Healthy Lifestyle Moderates Attributable Risk of All-Cause, Cardiovascular, and Premature Death Due to NAFLD

|  | **Group** | **HR (95%CI)** | **AR%** | **Adjustment ratio for AR%** |
| --- | --- | --- | --- | --- |
| **All-cause mortality** |  |  |  |  |
| NAFLD | No | 1(ref) |  |  |
|  | Yes | 1.09(0.93,1.28) | 9.3% |  |
| Adjusted by Depression | Yes | 1(ref) |  |  |
|  | No | 1.06(0.90,1.24) | 5.9% | 36.6% |
| Adjusted by Smoking | Yes | 1(ref) |  |  |
|  | No | 1.08(0.92,1.26) | 7.6% | 19.4% |
| Adjusted by Vigorous PA | No | 1(ref) |  |  |
|  | Yes | 1.03(0.88,1.20) | 3.0% | 67.7% |
| Adjusted by Healthy sleep | No | 1(ref) |  |  |
|  | Yes | 1.08(0.93,1.27) | 8.3% | 10.8% |
| Adjusted by Healthy diet | No | 1(ref) |  |  |
|  | Yes | 1.06(0.91,1.23) | 6.0% | 35.5% |
| Adjusted by Healthy lifestyle scores | 0-2 | 1(ref) |  |  |
|  | 3-5 | 1.02(0.87,1.19) | 1.8% | 80.6% |
| **Cardiovascular mortality** |  |  |  |  |
| NAFLD | No | 1(ref) |  |  |
|  | Yes | 1.24(0.97,1.58) | 24.1% |  |
| Adjusted by Depression | Yes | 1(ref) |  |  |
|  | No | 1.20(0.94,1.55) | 20.4% | 15.4% |
| Adjusted by Smoking | Yes | 1(ref) |  |  |
|  | No | 1.24(0.97,1.57) | 23.6% | 2.1% |
| Adjusted by Vigorous PA | No | 1(ref) |  |  |
|  | Yes | 1.17(0.92,1.49) | 17.1% | 29.0% |
| Adjusted by Healthy sleep | No | 1(ref) |  |  |
|  | Yes | 1.23(0.97,1.56) | 23.1% | 4.1% |
| Adjusted by Healthy diet | No | 1(ref) |  |  |
|  | Yes | 1.21(0.94,1.55) | 20.8% | 13.7% |
| Adjusted by Healthy lifestyle scores | 0-2 | 1(ref) |  |  |
|  | 3-5 | 1.19(0.92,1.53) | 18.8% | 22.0% |
| **Premature death** |  |  |  |  |
| NAFLD | No | 1(ref) |  |  |
|  | Yes | 1.72(1.20,2.46) | 71.8% |  |
| Adjusted by Depression | Yes | 1(ref) |  |  |
|  | No | 1.72(1.19,2.47) | 71.6% | 0.3% |
| Adjusted by Smoking | Yes | 1(ref) |  |  |
|  | No | 1.71(1.19,2.45) | 71.0% | 1.1% |
| Adjusted by Vigorous PA | No | 1(ref) |  |  |
|  | Yes | 1.59(1.12,2.26) | 59.0% | 17.8% |
| Adjusted by Healthy sleep | No | 1(ref) |  |  |
|  | Yes | 1.73(1.22,2.46) | 72.8% | -1.4% |
| Adjusted by Healthy diet | No | 1(ref) |  |  |
|  | Yes | 1.55(1.11,2.17) | 54.9% | 23.5% |
| Adjusted by Healthy lifestyle scores | 0-2 | 1(ref) |  |  |
|  | 3-5 | 1.64(1.15,2.33) | 63.6% | 11.4% |

Note: Model was adjusted for age, gender, ethnicity, marital status, education and poverty status, BMI, cardiovascular disease, and cancer. Abbreviation: NAFLD, nonalcoholic fatty liver disease; HR, hazard ratio; CI, confidence interval; AR, Attributable Risk; ref, reference; PA, physical activity.
