# Supplementary figures and images for: Association between Lifestyle Modification and All-Cause, Cardiovascular, and Premature Mortality in Individuals with Non-Alcoholic Fatty Liver Disease
Source: Nutrients. 2024 Jun 28;16(13):2063. doi: 10.3390/nu16132063 (PMC11243540; doi:10.3390/nu16132063)

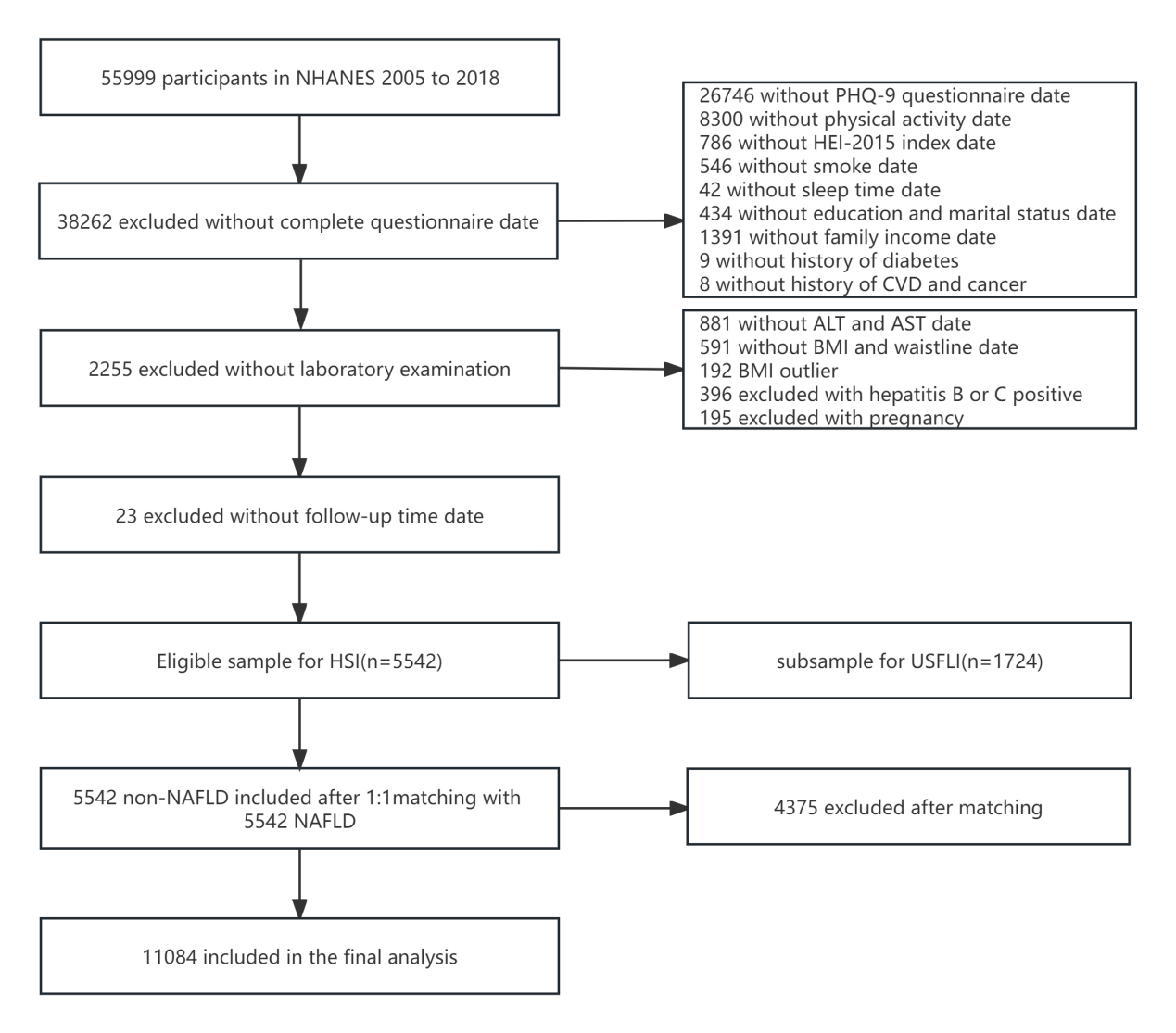

Supplement: Supplementary file 1 [file nutrients-16-02063-s001.zip › Supplementary materials/Figure S1.png]

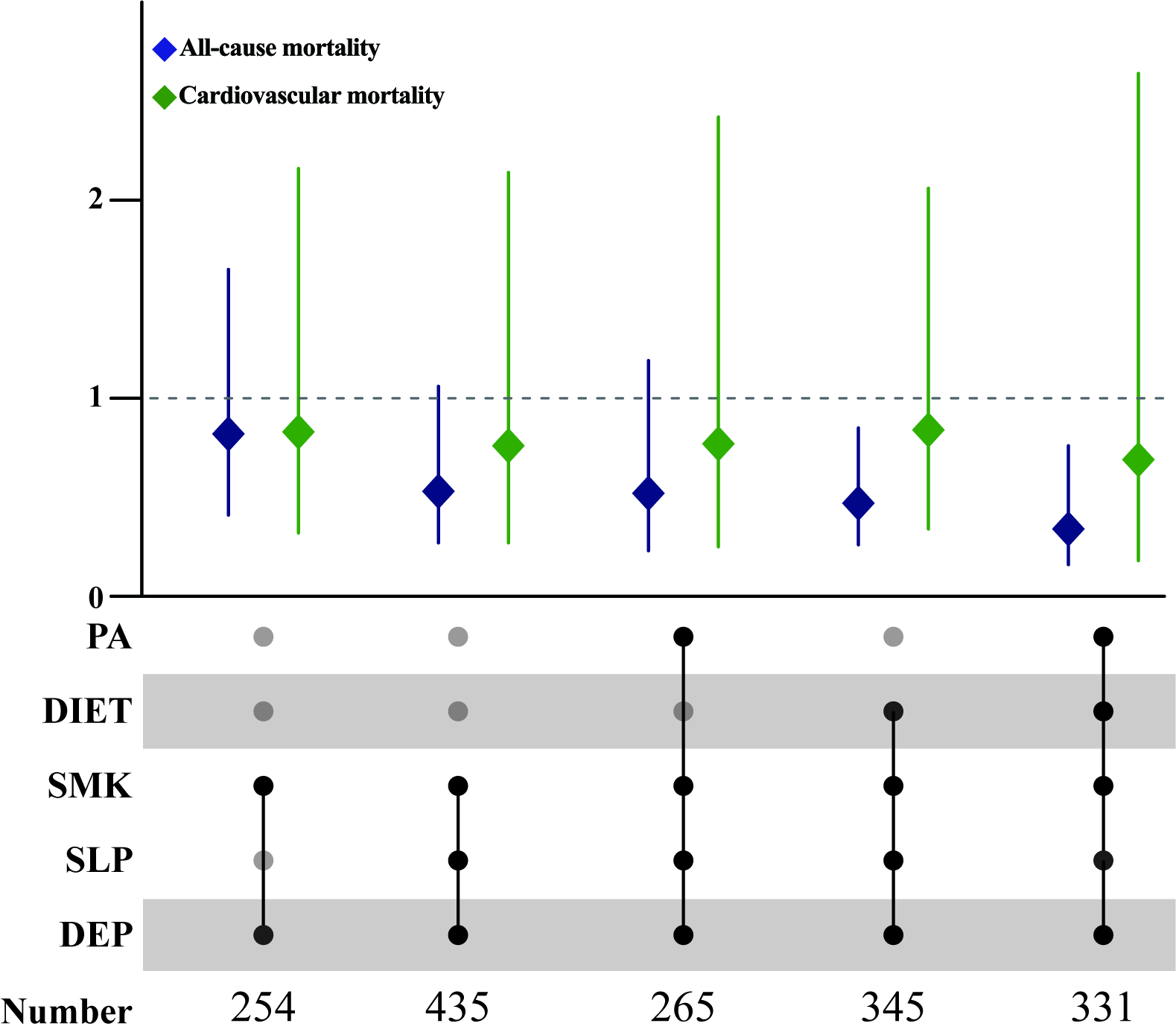

Supplement: Supplementary file 1 [file nutrients-16-02063-s001.zip › Supplementary materials/Figure S10.tif]

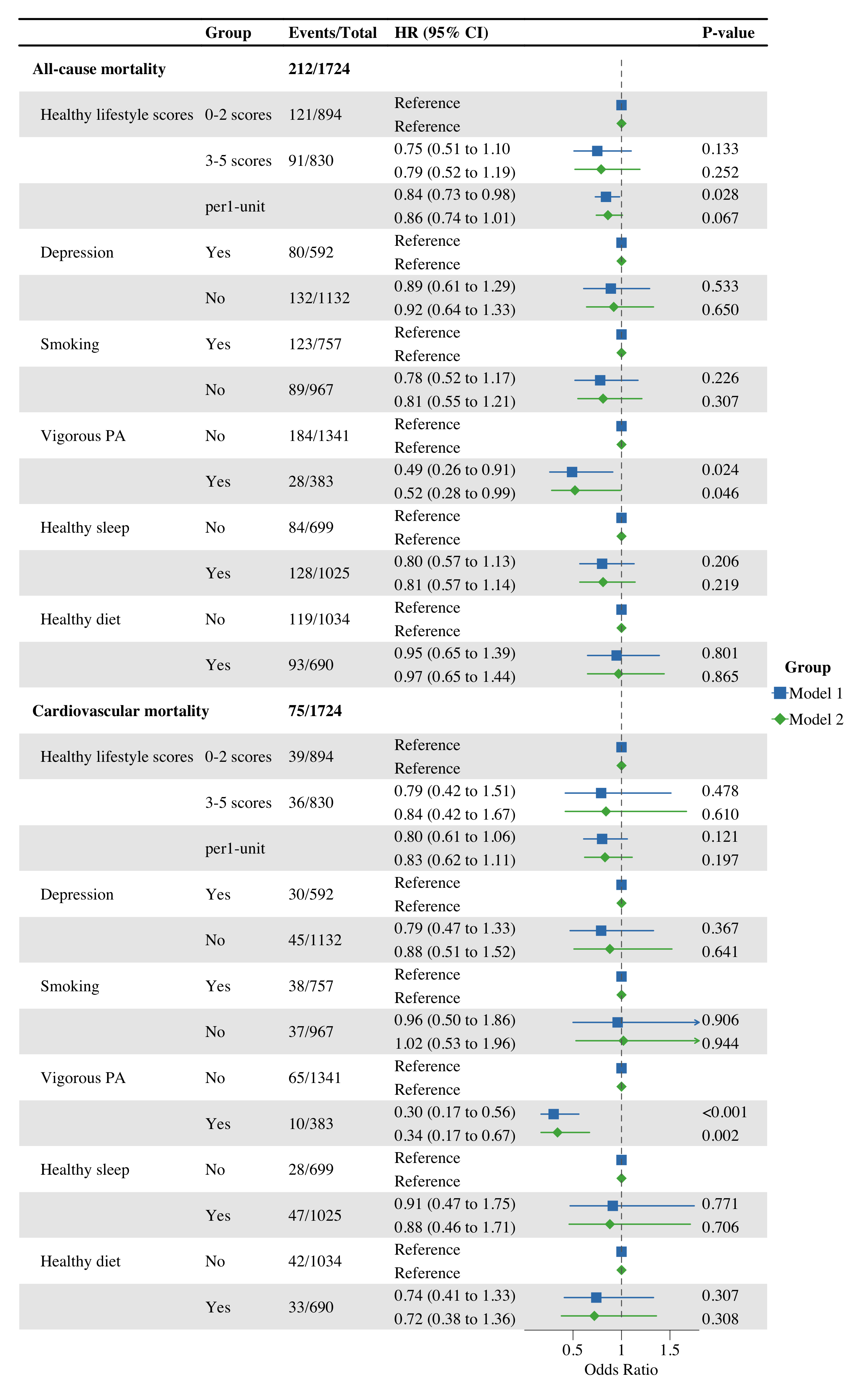

Supplement: Supplementary file 1 [file nutrients-16-02063-s001.zip › Supplementary materials/Figure S11.tiff]

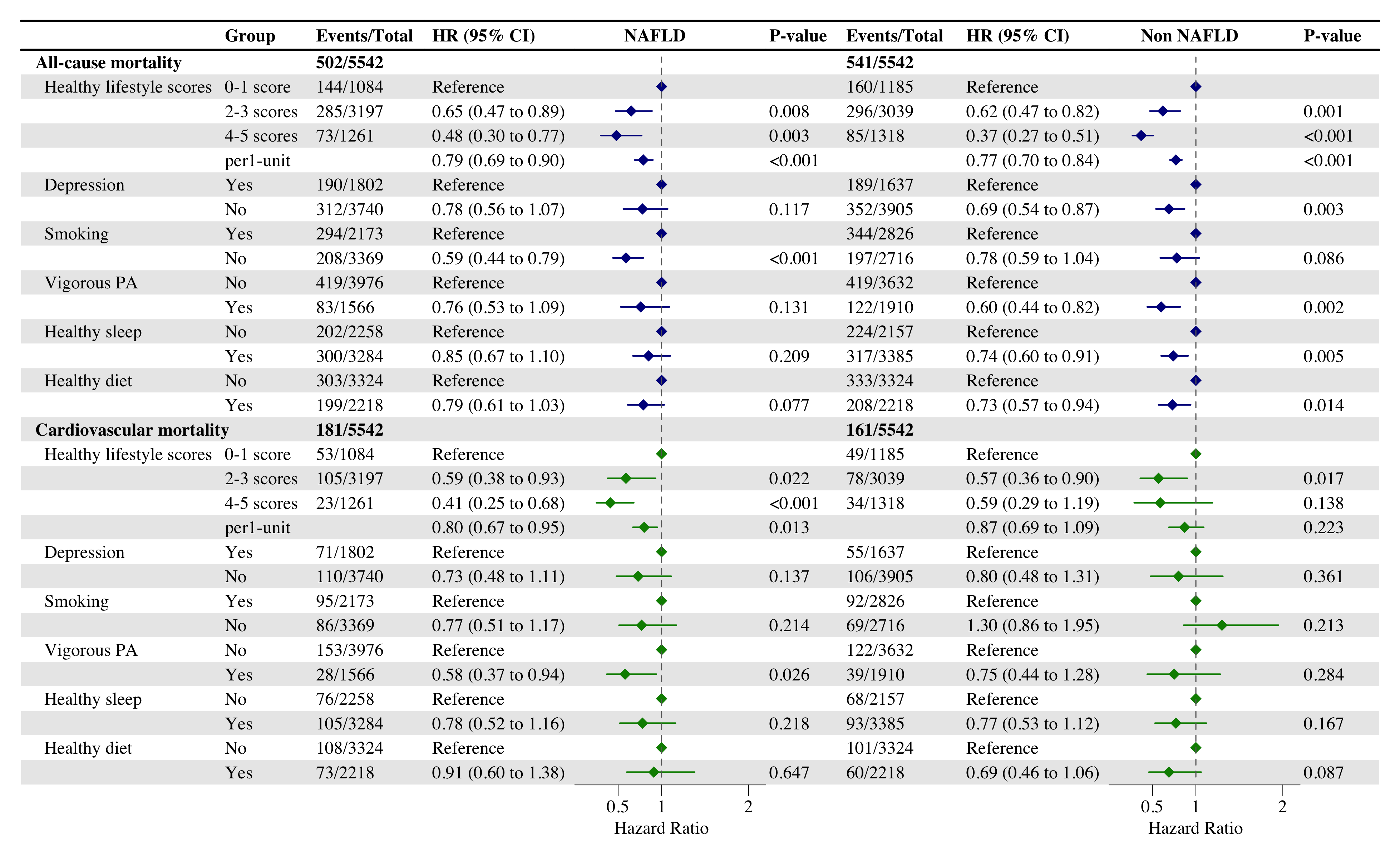

Supplement: Supplementary file 1 [file nutrients-16-02063-s001.zip › Supplementary materials/Figure S2.tiff]

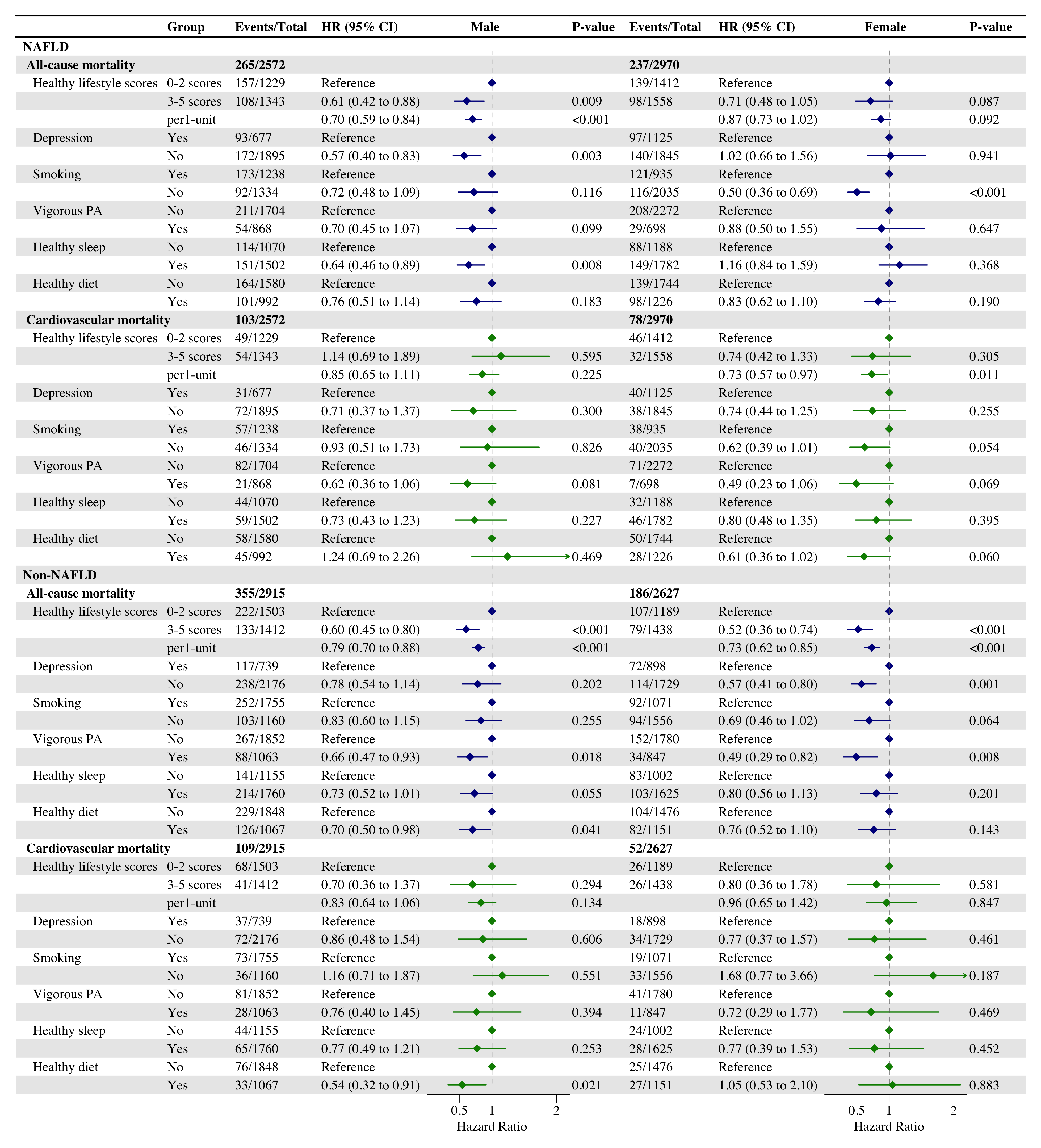

Supplement: Supplementary file 1 [file nutrients-16-02063-s001.zip › Supplementary materials/Figure S3.tif]

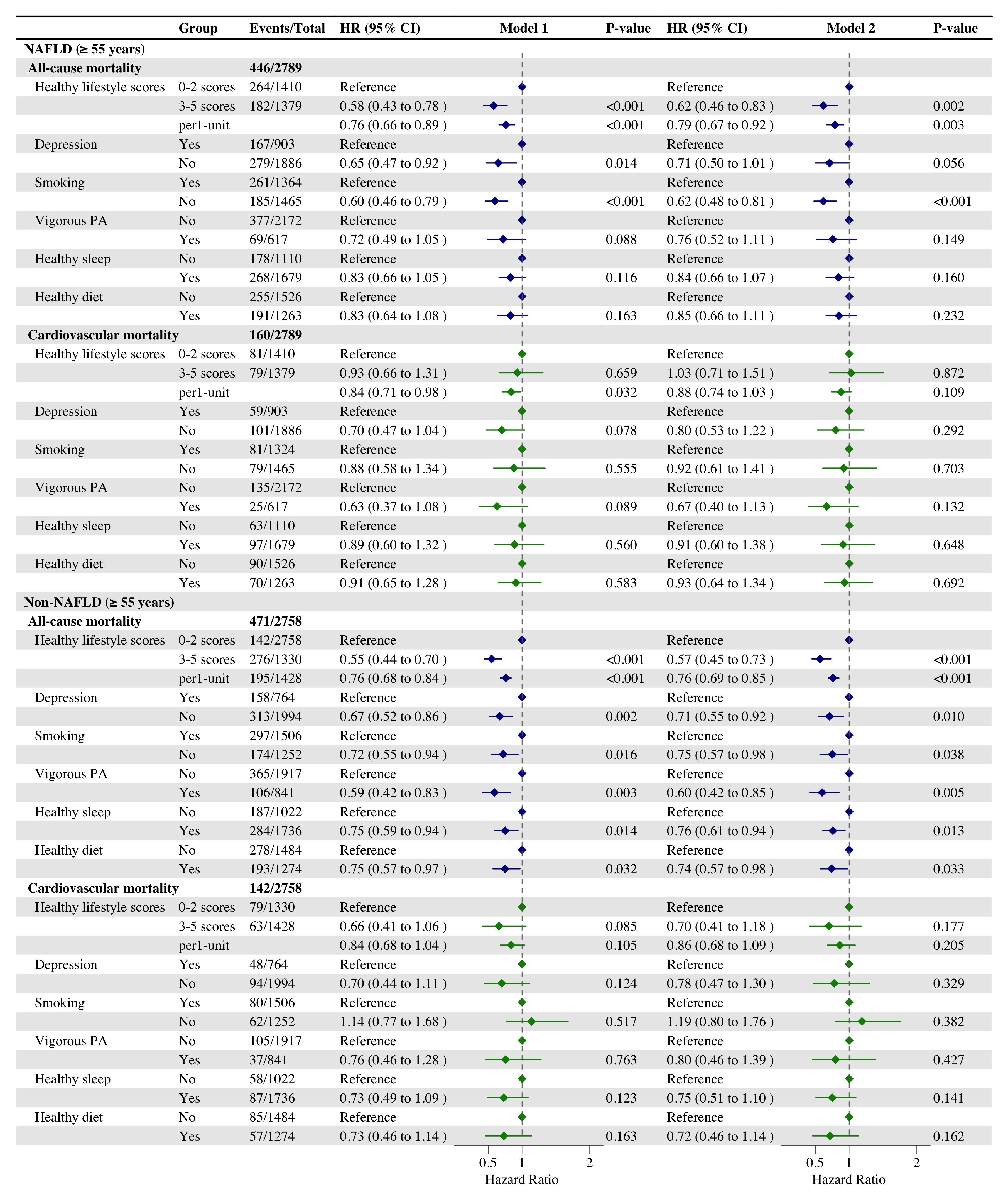

Supplement: Supplementary file 1 [file nutrients-16-02063-s001.zip › Supplementary materials/Figure S4.jpg]

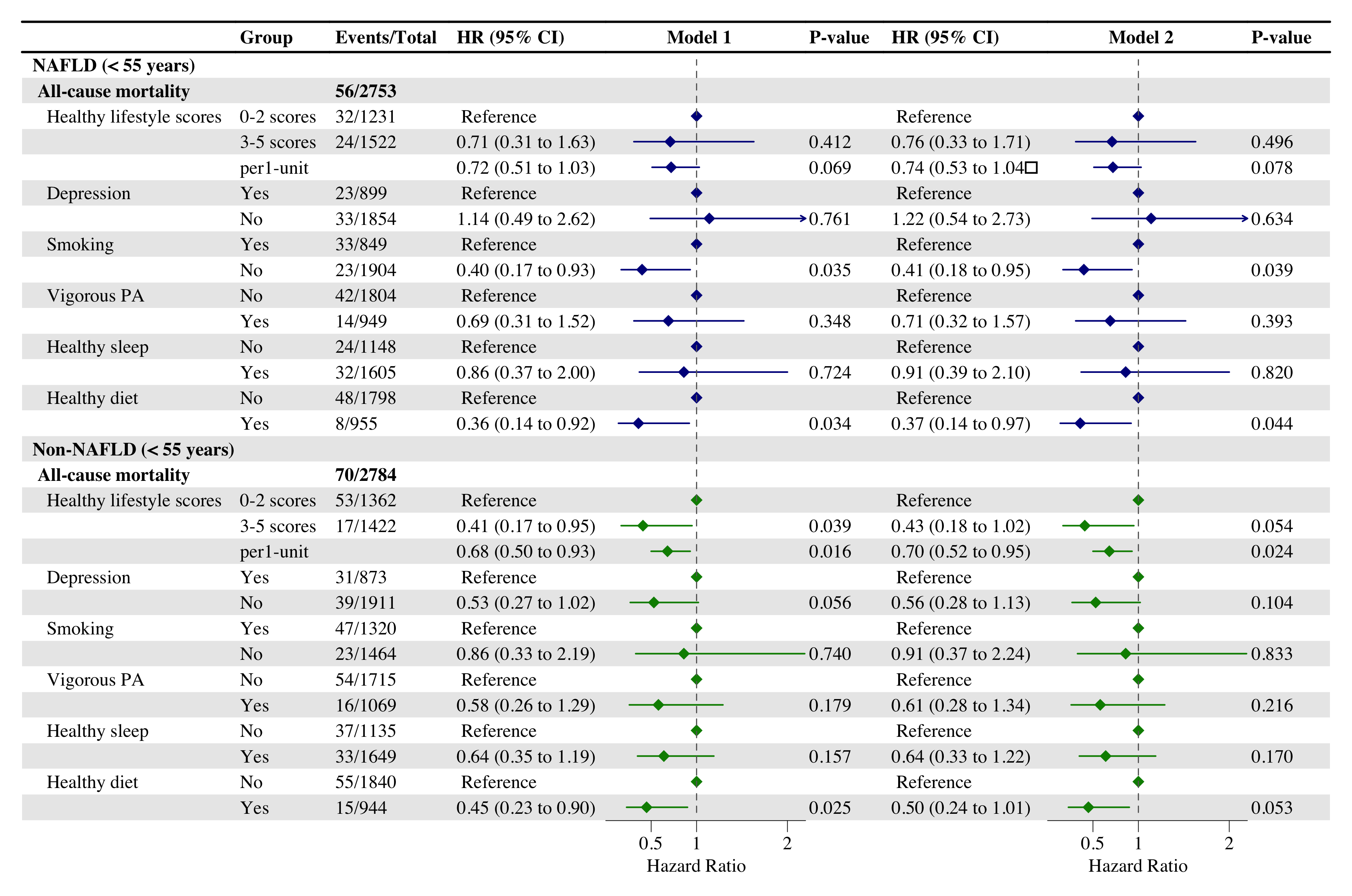

Supplement: Supplementary file 1 [file nutrients-16-02063-s001.zip › Supplementary materials/Figure S5.tiff]

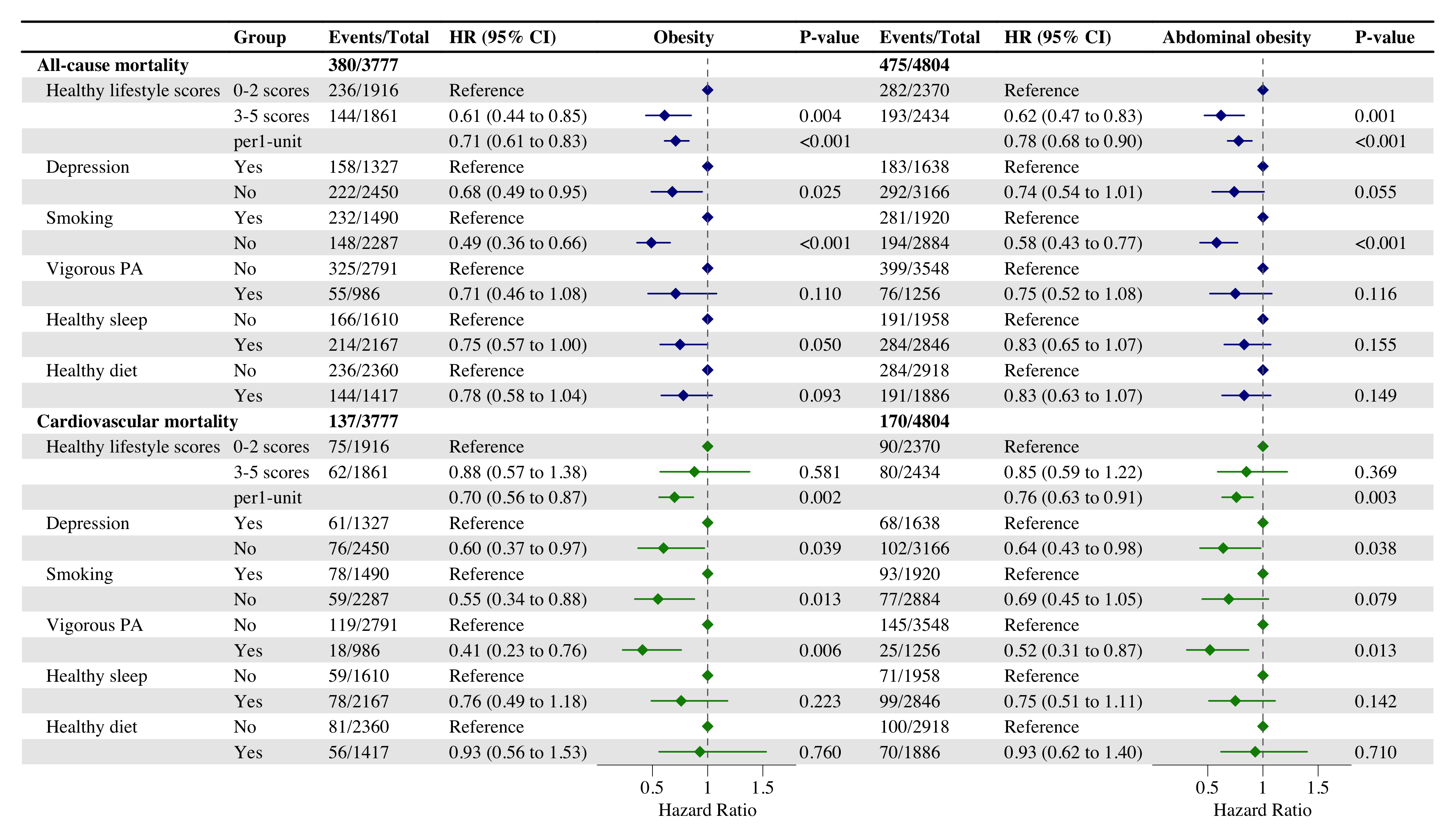

Supplement: Supplementary file 1 [file nutrients-16-02063-s001.zip › Supplementary materials/Figure S6.tiff]

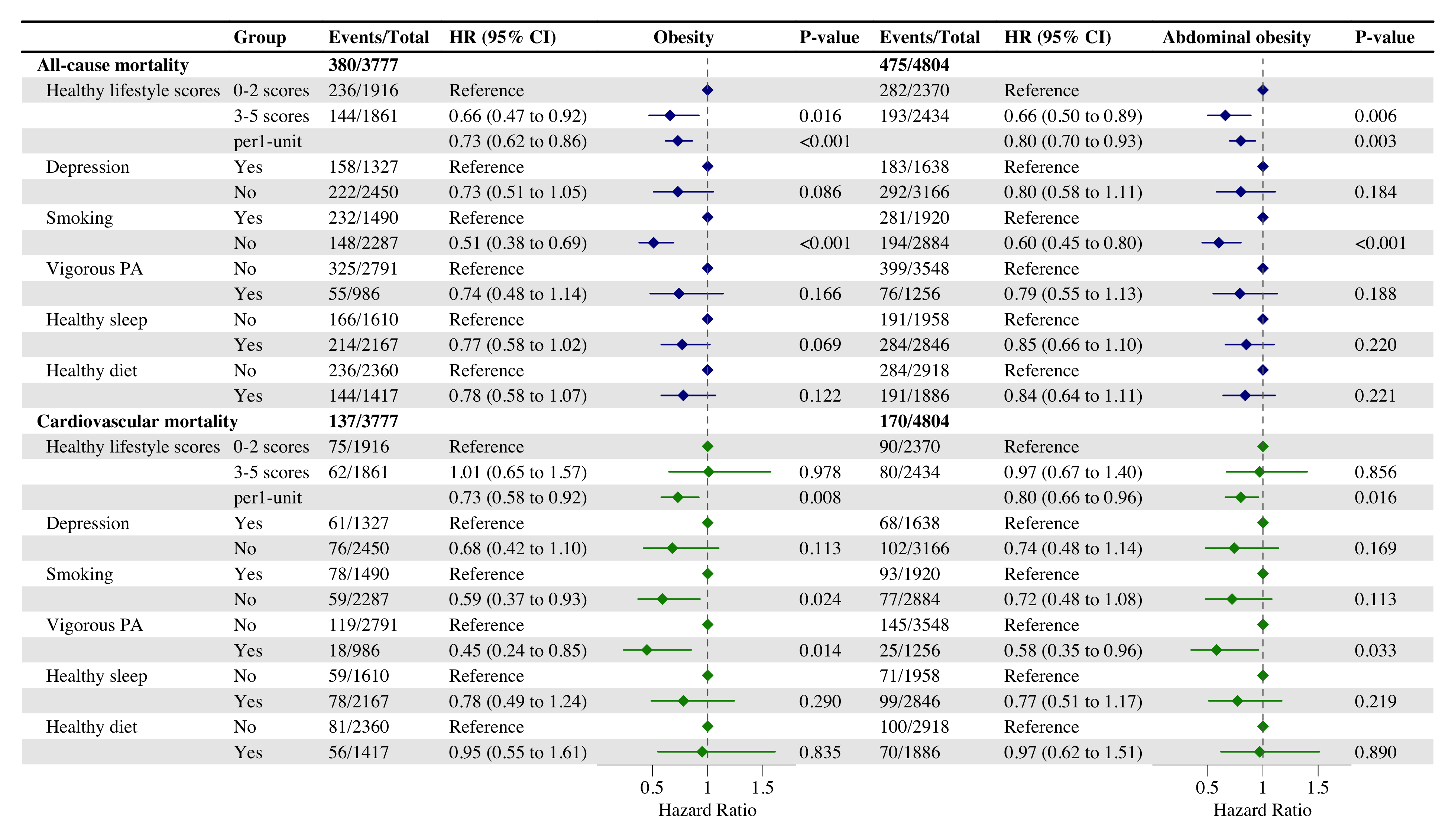

Supplement: Supplementary file 1 [file nutrients-16-02063-s001.zip › Supplementary materials/Figure S7.tiff]

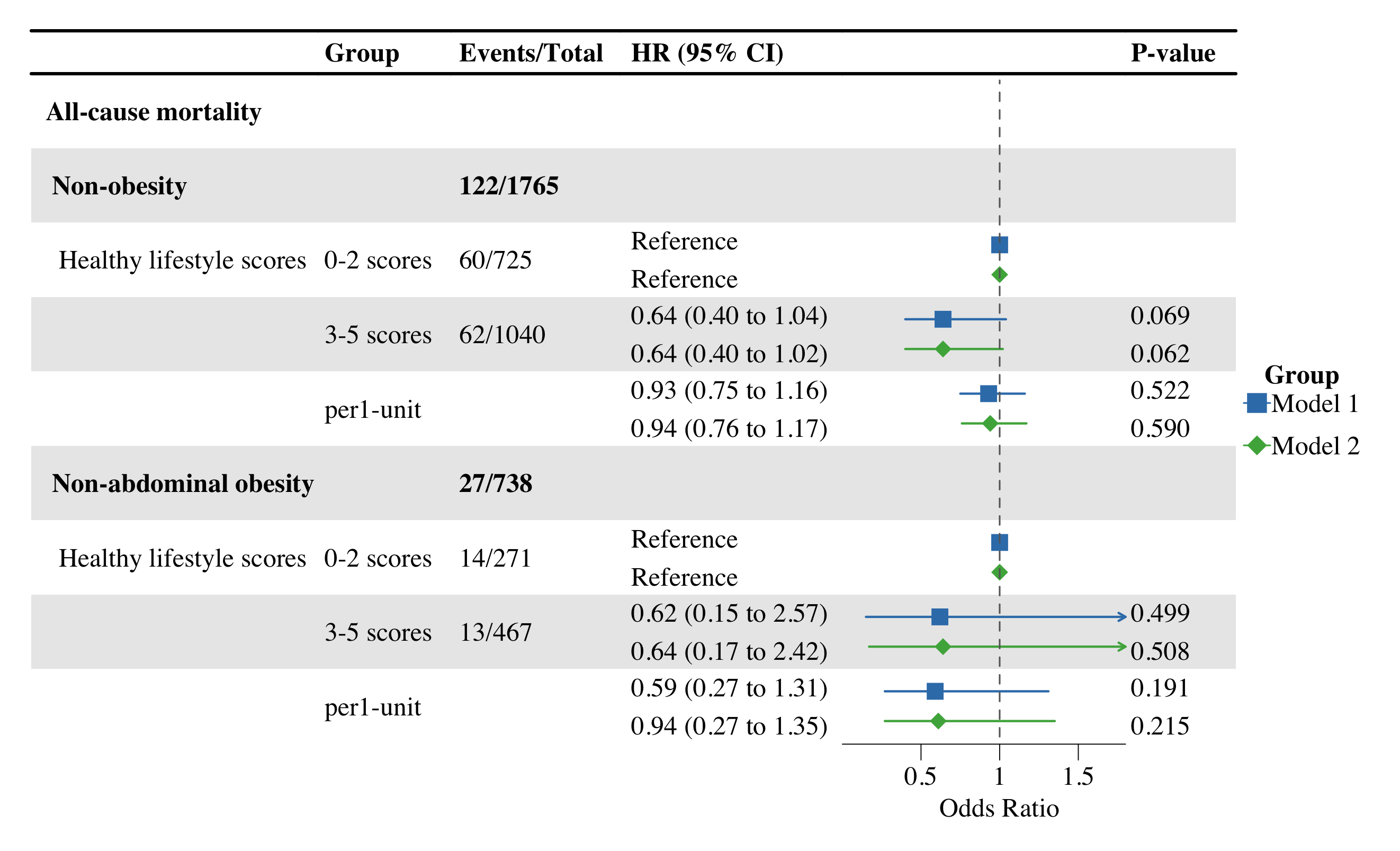

Supplement: Supplementary file 1 [file nutrients-16-02063-s001.zip › Supplementary materials/Figure S8.tiff]

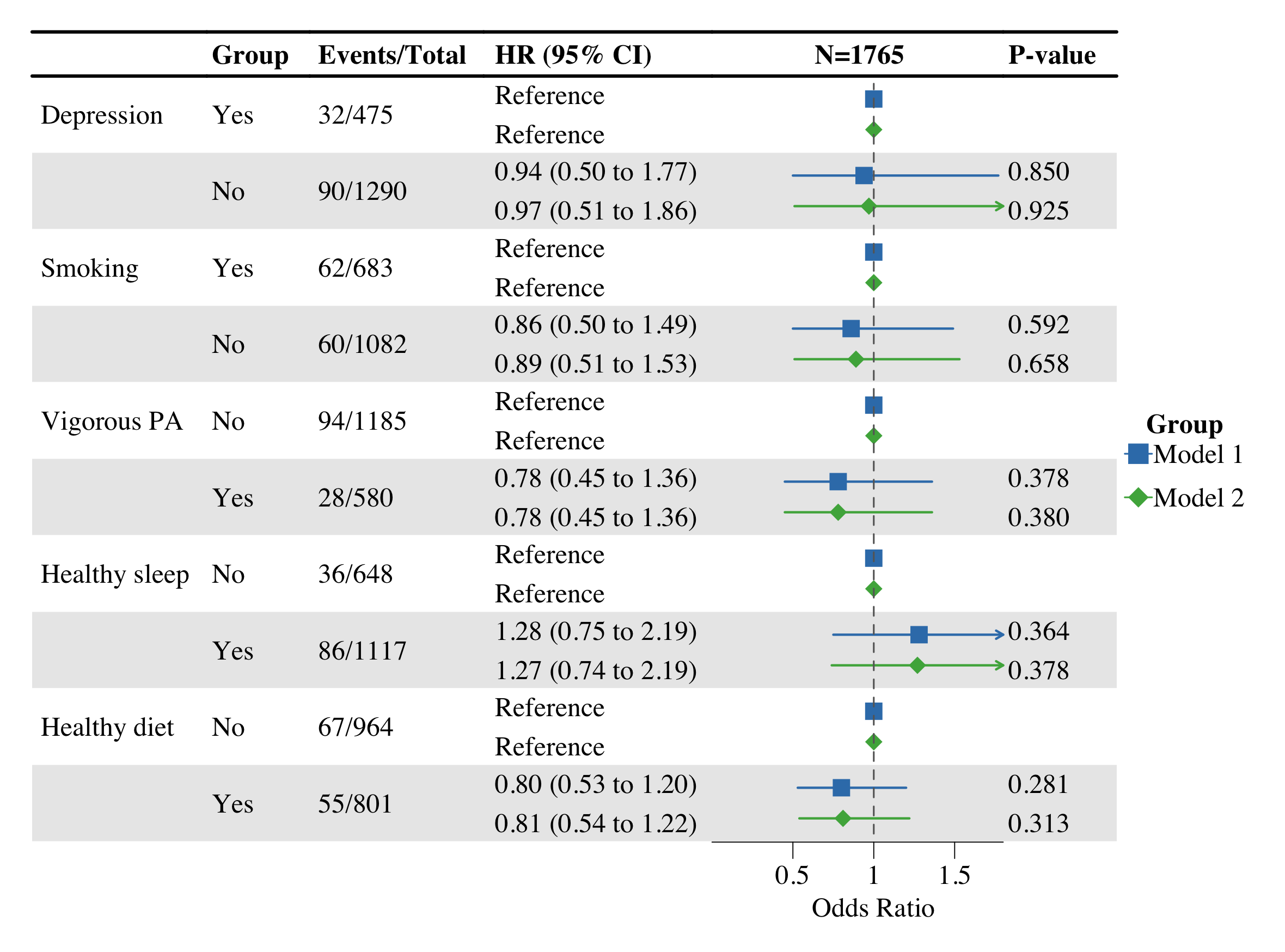

Supplement: Supplementary file 1 [file nutrients-16-02063-s001.zip › Supplementary materials/Figure S9.tiff]
